# Self‐reported urinary tract infection and bacterial vaginosis symptoms among indigenous adolescents during seasonal periods of water scarcity: A cross‐sectional study in Bandarban Hill District of Bangladesh

**DOI:** 10.1002/hsr2.2107

**Published:** 2024-05-06

**Authors:** Plabon Sarkar, M. A. Rifat, Imdadul Haque Talukdar, Nobonita Saha, Nicole S. Rodriguez Neufeld, Md. Ibrahim Miah, Sanjib Saha

**Affiliations:** ^1^ Caritas Bangladesh Dhaka Bangladesh; ^2^ Department of Global Public Health Karolinska Institutet Stockholm Sweden; ^3^ Department of Learning, Informatics, Management and Ethics (LIME) Karolinska Institutet Stockholm Sweden; ^4^ Institute of Nutrition and Food Science University of Dhaka Dhaka Bangladesh; ^5^ Department of Microbiology University of Dhaka Dhaka Bangladesh; ^6^ Health Economics Unit, Department of Clinical Sciences Lund University Lund Sweden

**Keywords:** adolescents, bacterial vaginosis, Bandarban, Bangladesh, Chattogram Hill Tracts, menstrual hygiene, urinary tract infection, water pollution, water scarcity

## Abstract

**Background and Aims:**

Water scarcity and poor water quality could lead to suboptimum menstrual hygiene practices, and subsequently urinary tract infection (UTI) and bacterial vaginosis (BV). In this study, we estimate the prevalence of self‐reported UTI and BV among indigenous adolescent girls during the water scarcity period in the Bandarban Hill Districts in south‐eastern Bangladesh.

**Methods:**

Using a cross‐sectional design, a total of 242 indigenous adolescent girls were selected and interviewed during the seasonal water scarcity period (from February to May 2022) in Bandarban. The difference in prevalence of any self‐reported UTI or BV symptoms by respondents' characteristics was assessed by *χ* ^2^ test. Multivariable logistic regression model was used to observe the associated factors.

**Results:**

The prevalence of self‐reported UTI, BV, and any symptoms of UTI or BV among the respondents were 35.54%, 28.93%, and 43.80%, respectively. Ethnicity, studentship status, source of water used for menstrual hygiene, and perceived water quality were significantly associated with the prevalence of any self‐reported UTI or BV symptoms.

**Conclusion:**

Findings recommend further research to cross‐check the validity of self‐reported prevalence and investigate if the episodes of UTI or BV could be attributable to water scarcity and poor water quality in study areas during dry period.

## 1 INTRODUCTION

Urinary tract infection (UTI) and bacterial vaginosis (BV) are caused by bacterial presence in the urinary tract and the vagina.^1^, ^2^ Factors, including water accessibility,^3^ water quality,^4^, ^5^, ^6^ and poor menstrual hygiene practices can contribute to the increased susceptibility to UTI and BV.^7^, ^8^, ^9^ BV is one of the most prevalent infections among women of reproductive age worldwide.^10^ Globally, 23%–29% of women of reproductive age suffer from BV at least once in their lifetime.^10^ On the other hand, around 50%–60% of adult women suffer UTI at least once in their lifetime.^11^

Lack of access to safe water is a global problem, with people living in water scarce areas, such as hillsides and deserts, being the worst victims of it.^12^ In Bangladesh, 98% of people have access to water supply, also the quality of water is debatable.^13^ It was reported that 80% of private piped water taps and majority of the pond water in Bangladesh was contaminated with *E. coli* bacteria.^13^ Indigenous people in Bangladesh are one of the most deprived population groups considering water accessibility.^14^ For example, only 56.8% indigenous people living in Bandarban district had access to basic drinking water sources.^15^ In the Chattogram division, there are three Hill Districts, namely Bandarban, Rangamati, and Khagrachari, which are resided by around 11 indigenous groups with a population totaling to 890,435.^16^, ^17^ Although tube‐well is the most common water source in Bangladesh, only 10.8% indigenous people in Bandarban have access to tube‐well water whereas more than half (59.4%) of them use spring water and 36% use surface water for meeting their daily needs.^18^ Furthermore, water scarcity is reported to be an attributable factor of poor water, sanitation, and hygiene (WASH) practices among more than half of the indigenous people living in Bandarban.^18^

The Shankha (Sangu), Matamuhuria, and Bakkhali are the main rivers of this region, and significant sources of surface water in Bandarban.^19^ During the dry season (January to May), indigenous people severely suffer from water crisis as major water sources dry up and underground water levels fall, consequently leading many families to migrate from one village to another.^17^, ^20^ Furthermore, many families have to collect water from distant streams, dying waterfalls, and rivers. This water is often contaminated by microorganisms contributing to hygiene poverty, and this forces girls to apply menstrual coping strategies.^21^

Indigenous adolescent girls in Bandarban experience severe water scarcity in the dry season which could lead to poor menstrual hygiene practices, and subsequently UTI and BV. While the impact of water scarcity on agriculture, food system, and WASH practices is often investigated, its impact on urogenital infection is often unaddressed. Studies conducted in India reported that poor menstrual hygiene might contribute to women's susceptibility to UTIs and BV.^9^, ^22^ Another study conducted in Ethiopia found that good menstrual hygiene was associated with access to water, and that restricted or no access to water can result in poor menstrual hygiene.^23^ However, despite being a country with high climate change vulnerability and increased susceptibility to water crisis, no study was conducted in Bangladesh to observe the prevalence of urogenital infection among adolescent girls in the areas experiencing seasonal water scarcity in Bangladesh. This study, therefore, aims to observe self‐reported UTI and BV prevalence among indigenous adolescent girls in Bandarban during the period of water scarcity. Adolescent girls were targeted considering their high vulnerability in terms of menstrual hygiene management, as compared with adult women who are more experienced in coping strategies and less accessible because of their involvement in agricultural and other livelihood activities. The study objective is to observe the prevalence of self‐reported UTI or BV prevalence and associated factors among indigenous adolescent girls in Bandarban district of Bangladesh.

This study is of critical importance as it sheds light on the possible impact of clean water accessibility on healthy menstrual practices in a vulnerable population. Further research should aim to explore whether environmental exposures, such as water scarcity and pollution, can be attributable to an increased risk of gynecological infections. This is of great importance given the impact of climate change on access to sufficient and clean water globally. In the case of many small indigenous groups, geographic and social marginalization, discrimination, and reliance on environmental surroundings, among others, can exponentially exacerbate the risk for gynecological infections, and potentially limit access or use of medical services for treatment.

## 2 MATERIALS AND METHODS

In this study, STROBE was considered as the reporting tool (Supporting Information File).^24^ Furthermore, the SAMPL guidelines^25^ and the guidelines developed by Assel et al.^26^ were considered to report statistical methods used and findings, respectively.

### 2.1 Settings

Bandarban is a district of the Chittagong administrative division located in south‐eastern Bangladesh. It is one of the three Hill Districts of Bangladesh, identified as the hardest to reach and has the lowest population density in the country. The population density is 87 per square kilometer (km) in Bandarban,^27^ whereas the national average is 976 per square km.^16^ Bandarban is characterized by some highest elevations in Bangladesh and 11 indigenous communities, who live in clusters all over the district, alongside mainstream Bengali people.^28^ Each of these indigenous groups has their own culture but they experience discrimination that result in their poor health status.^29^ There are 436,950 people living in Bandarban of which 173,351 are less than 18 years of age.^27^ Among the under 18 population, 142,401 (44.39%) belong to indigenous communities.^19^ It consists of seven subdistricts, locally called Upazila, and 29 unions (smaller administrative area). Majority of the indigenous people live in small villages, called Para, remotely located in hillslopes, and near to springs, rivers, and valleys. In our study, Bandarban district was considered because the severity of water scarcity in Bandarban was higher than the other two districts as reported in mass media.^20^

### 2.2 Study design and sampling

The design of this study was cross‐sectional. The study population included those living in areas where people experience water scarcity during the dry season in Bandarban. The study area was selected with the help of local communities while outlining the study plan. Eligibility criteria included the following: participants should be adolescent girls between the ages of 10–19, from indigenous communities living in areas experiencing water scarcity during dry seasons in Bandarban. At a population level, there was no information about the prevalence of UTI and/or BV among adolescent girls in Bandarban. However, a study conducted in Sylhet, a north‐east district of Bangladesh, found that the prevalence of UTI among rural pregnant women was 8.9%.^30^ We assumed a similar prevalence of UTI in our target population. Therefore, sample size estimation was conducted by considering a population proportion (*p*) of 0.089, 95% confidence level (C.I.), 5% significance level (*d*), and 20% nonresponse rate. The formula used to calculate the sample size was: *n* = *p*(1‐*p*)*z/*d* ^2^, where *n *= sample size, *z* = 1.96 at 95% C.I. Considering 20% nonresponse, the calculation showed a total of 150 eligible participants to be interviewed. In this study, 242 eligible participants were conveniently selected and interviewed. This sample size justified the number of respondents required to draw statistical inference even if the population proportion was 15.5%. The response rate was 100%.

### 2.3 Interview and data management

**Figure 1:**
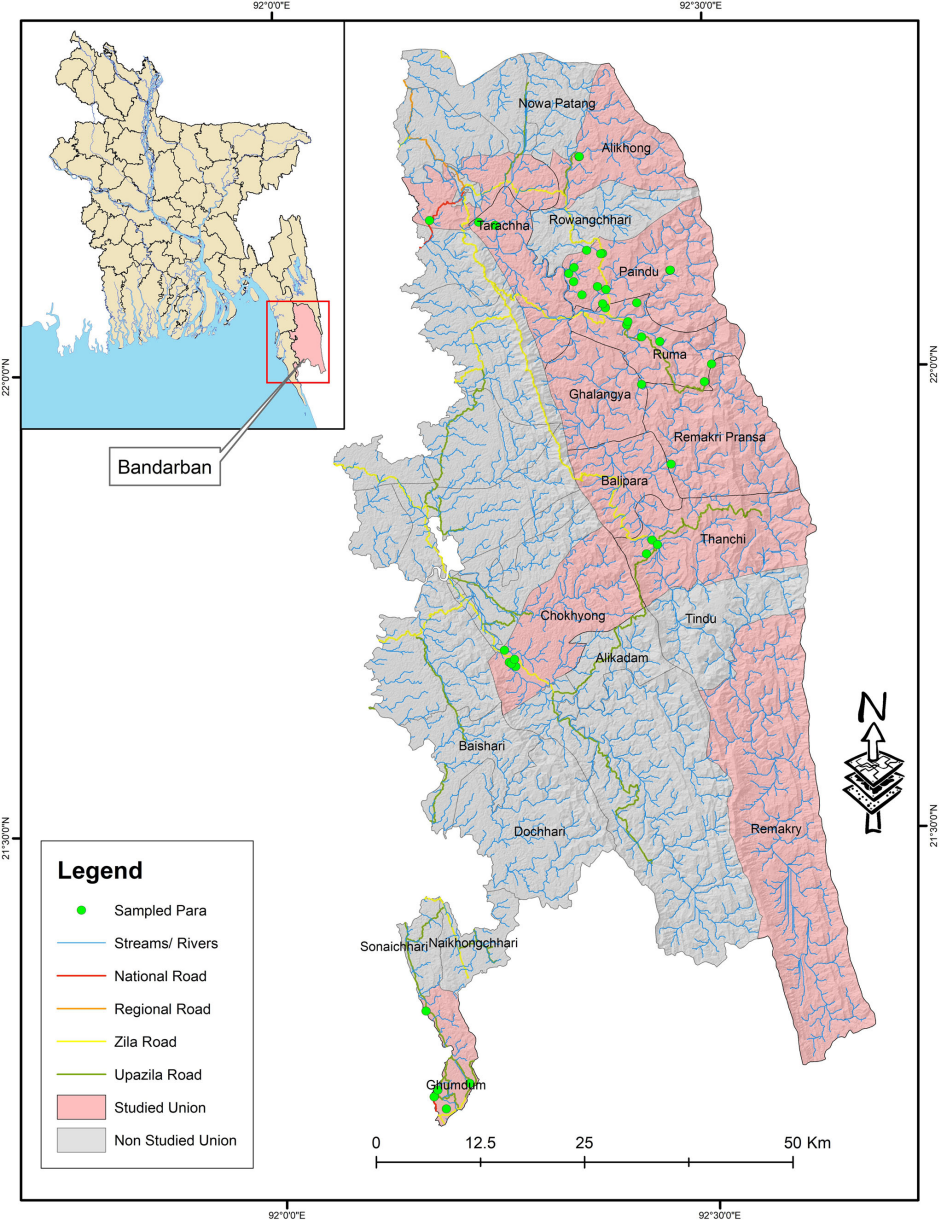
Location of the data collection sites.

Data collection was conducted in 38 Paras from 11 unions of the six subdistricts of Bandarban from February to May 2022 (Figure 1). No rainfall was recorded during this period. Data was collected through face‐to‐face interviews using questionnaires. The questionnaires were translated into both Bengali and Marma languages. The interviews were conducted by female interviewers who were trained in data collection and belong to the indigenous communities. They were from the Marma, Tripura, and Tanchangya communities and native speakers of the respective indigenous languages. The questionnaire was transcribed into an SPSS datasheet, and the responses were entered. Each questionnaire was coded with an identity number (ID number) so that data can be depersonalized during analysis. Both the printed questionnaire and soft copy of the data set were stored, and privacy of the individual information was strictly maintained.

### 2.4 Covariates

The questionnaire consisted of five sections, sociodemographic information, water sources, menstrual hygiene practices, perceived difficulties in menstrual hygiene practices due to water scarcity, and self‐reported UTI and BV symptoms. Among the sociodemographic characteristics, age, marital status, studentship status, level of education, family size, and ethnicity were considered. Information about water sources included sources of water for family use, water source for maintaining menstrual hygiene practice, time required to collect water, and history of family relocation in the last 5 years due to water scarcity. The menstrual hygiene practices included information about number of genital wash and bathing per day and perceived water quality during last menstrual period. Perceived quality of water used in the last menstrual period was measured using a 5‐point Likert scale‐based items, considering 1 = *very good/excellent*, 2 = *good*, 3 = *average/okay*, 4 = *poor*, 5 = *very poor*. During analysis, however, this variable was converted into two categories, including 0 = “good” (responses 1–3 of original scale) and 1 = “bad” (responses 4–5 of original scale). Perceived difficulty in menstrual hygiene practices consisted of five items. These items included difficulties faced in the last menstruation period due to, (1) distance of water sources from residence, (2) washing genitals due to water scarcity, (3) bathing due to water scarcity, (4) cleaning clothes, absorbents, and other relevant materials due to water scarcity and (5) cleaning hands before and after changing absorbents due to water scarcity. For each of the items, a 5‐point Likert scale was established, ranging from 0 to 4 where 0 = *never*, 1 = *occasionally/seldom*, 2 = *sometimes*, 3 = *often*, and 4 = *always*. The scale reliability and validity were measured by Cronbach's *⍺* and Rasch analysis and detailed out elsewhere.^31^ In this case, the value of Cronbach's *⍺* was 0.91 indicating a high internal validity. Subsequently, a perceived difficulty score was calculated for each of the respondents. For ease of analysis and interpretation, other continuous variables such as age, time to collect water, number of family members, and perceived difficulty score were converted into categorical variables. The outcome variables were binary, looking at self‐reported symptoms for either UTI or BV. Responses concerning having symptoms of UTI, BV or both were coded as 1 (any symptom), and with neither of these symptoms were coded as 0 (no symptom).

For UTI, the following symptoms were considered: (a) feeling pain or burning while urinating, (b) frequent urination, (c) feeling the need to urinate despite having an empty bladder, (d) blood in urine and pressure or cramping in the groin or lower abdomen.^32^ Symptoms including (a) a thin white or gray vaginal discharge, (b) feeling pain, itching, or burning in the vagina, (c) a strong fish‐like odor, especially after sex, (d) burning when peeing, and (e) itching around the outside of the vagina^33^ was considered for BV. Finally, the symptoms were crosschecked by local physicians dealing with illnesses of adolescent girls and reproductive women.

### 2.5 Data analysis

Distribution of the participants' characteristics, by no symptom versus any symptom, was observed using crosstabs. The difference in proportion between no symptom and any symptom categories by participants' characteristics was estimated using *χ* ^2^ test. Unadjusted logistic regression was performed to observe which variables are significantly associated with an event of either UTI or BV. Variables which showed a significant association in the unadjusted model or were likely to have effect on outcome were included in the adjusted model. All the statistical tests were two‐tailed and performed at 95% confidence level considering *p* value < 0.05 as statistically significant. Data analysis was conducted using Stata software (version 17 Stata Corp).

### 2.6 Ethics approval

The study was conducted in accordance with the principles mentioned in the Declaration of Helsinki. Ethical Review Committee of the Faculty of Biological Sciences, University of Dhaka, Bangladesh approved the study (reference number: 176/Biol. Scs.). Before interviewing, every participant and their parents/legal guardians were clearly explained about the objectives of the study and informed consent was received. Before analysis, the personal/individual level data was anonymized and dealt with maintaining confidentiality.

## 3 RESULTS

### 3.1 Characteristic of participants

The sociodemographic characteristics of the participants in this study are presented in Table 1. More than half (60.33%) of the adolescents had age range 16–19 years. Ethnicity of participants was represented with 32.23% Marma, 33.06% Tripura, 20.66% Tanchangya, and 14.02% from other ethnic communities such as Bawm, Chak, and Lushai. Most of them were students (81.82%), and the rest (18.18%) were engaged in agriculture, household activities, or other services.

**Table 1:** Characteristics of indigenous adolescent girls participated in this study (*n* = 242).

Characteristics	Frequency (Percentage)	No symptom	Any symptom	*p* Value
Age (years)				
10–15	96 (39.67)	70 (72.92)	26 (27.08)	<0.001
16–19	146 (60.33)	66 (45.21)	80 (54.79)	
Currently studying				
Yes	198 (81.82)	118 (59.60)	80 (40.40)	0.02
No	44 (18.18)	18 (40.91)	26 (59.09)	
Ethnicity				
Marma	78 (32.23)	18 (23.08)	60 (76.92)	<0.001
Tripura	80 (33.06)	60 (75.00)	20 (25.00)	
Tanchangya	50 (20.66)	33 (66.00)	17 (34.00)	
Others	34 (14.02)	25 (73.53)	09 (26.47)	
Number of family members				
<6	105 (43.39)	55 (52.38)	50 (47.62)	0.30
≥6	137 (56.61)	81 (59.12)	56 (40.88)	
Water source for family use				
Underground	37 (15.29)	18 (48.65)	19 (51.35)	0.02
Surface	89 (36.78)	42 (47.19)	47 (52.81)	
Spring	116 (47.93)	76 (65.52)	40 (34.48)	
Water source for menstrual hygiene				
Underground	43 (17.77)	23 (53.49)	20 (46.51)	0.001
Surface	88 (36.36)	37 (42.05)	51 (57.95)	
Spring	111 (45.87)	76 (68.47)	35 (31.53)	
Time required to collect water				
<20 min	135 (55.79)	55 (40.74)	80 (59.26)	<0.001
≥20 min	107 (44.21)	81 (75.70)	26 (24.30)	
Family relocation in last 5 years due to water scarcity				
No	231 (95.45)	132 (57.14)	99 (42.86)	0.12
Yes	11 (4.55)	4 (36.36)	7 (63.64)	
Water purification for menstrual use				
No	135 (55.79)	62 (45.93)	73 (54.07)	<0.001
Yes	107 (44.21)	74 (69.16)	33 (30.84)	
Frequency of genital wash per day				
≤4 times	143 (59.09)	83 (58.04)	60 (41.96)	0.49
>4 times	99 (40.91)	53 (53.54)	46 (46.46)	
Genital wash practices				
Water only	152 (62.81)	96 (63.16)	56 (36.84)	0.005
Soap and water	90 (37.19)	40 (44.44)	50 (55.56)	
Daily bathing during menstruation				
No	12 (4.96)	6 (50.00)	6 (50.00)	0.66
Yes	230 (95.04)	130 (56.52)	100 (43.48)	
Experienced water scarcity/shortage in last menstruation period				
No	111 (45.87)	41 (36.94)	70 (63.06)	<0.001
Yes	131 (54.13)	95 (72.52)	36 (27.48)	
Perceived difficulty score				
≤0.96 (average)	108 (44.63)	41 (37.96)	67 (62.04)	<0.001
>0.96 (average)	134 (55.37)	95 (70.90)	39 (29.10)	
Perceived quality of water during last menstrual period				
Good	169 (69.83)	76 (44.97)	93 (55.03)	<0.001
Bad	73 (30.17)	60 (82.19)	13 (17.81)	
Self‐reported prevalence of UTI symptom	86 (35.54)	–	–	–
Self‐reported prevalence of bacterial vaginosis symptom	70 (28.93)	–	–	–

Self‐reported prevalence of UTI and BV among participants were 35.54% and 28.93%, respectively. A total of 106 adolescent girls (43.80%) reported any symptoms of UTI and/or BV infection. Among respondents' families, 47.93% used water from spring/waterfall, 36.78% used surface water—such as ponds, wells, lakes, and river water, and 15.29% used underground water, for cleaning, washing, and bathing. Regarding menstrual hygiene, 45.87% adolescent girls used water from springs, 36.36% used surface water, and 17.77% used underground water. On average, 19.24 min were spent in collecting water from the nearest sources. Regarding, however, cleanliness, 69.83% perceived the quality of water as “not bad” despite the change in water quality. In addition, 54.13% of participants experienced water shortage during their last menstrual period, and 44.21% purified water by filtration, distillation, and boiling before engaging in menstrual hygiene practices. On average, the participants cleaned their genitals 4.24 times a day during menstruation. 62.81% reported using soap and water, and 37.19% used only water for cleaning genitals. Most of the participants (96.04%) bathed daily during menstruation. In the last 5 years, 4.55% of the respondents' families had to relocate their residences due to water scarcity.

The distribution of respondents with any self‐reported symptoms of UTI or BV and no symptom was significantly differed by age (*p* < 0.001), ethnicity (*p* < 0.001), time required to collect water (*p* < 0.001), perceived water quality during last menstruation (*p* < 0.001), source of water both for family use (*p* = < 0.05) and menstrual hygiene (*p* < 0.01), purification of the water for menstrual hygiene practices (*p* < 0.001), genital wash practices (*p* < 0.001), experienced water shortage during last menstrual period (*p* = 0.001), and perceived difficulty score (*p* < 0.001).

### 3.2 Factors associated with any self‐reported symptoms of UTI or BV

Table 2 represents the multivariable logistic regression model indicating the factors associated with any self‐reported UTI or BV symptoms among the participants. Multivariable logistic regression analysis showed that occupation, ethnicity, source of water for menstrual hygiene, and perceived quality of water used during the last menstrual period were significantly associated with any self‐reported symptoms of UTI or BV. Participants who were currently not students were seen to have 3.74 times more risk of having UTI and/or BV symptoms (95% CI: 1.23, 11.39) compared with those who were students, as expressed as adjusted odds ratio (AOR). The odds of having self‐reported UTI and/or BV symptoms were high among Marma adolescents compared with their counterparts, including Tripura (AOR = 0.75, 95% CI = 0.01, 0.59), Tanchangya (AOR: 0.08, 95% CI = 0.03, 0.27), and others (AOR = 0.07, 95% CI = 0.02, 0.30). Furthermore, respondents who used surface water (AOR = 2.92, 95% CI = 1.13, 7.52) and spring/waterfall water (AOR = 6.30, 95% CI = 1.40, 28.42) had higher risk of having self‐reported UTI and/or BV symptoms than those who used underground water. Finally, participants who perceived water quality as bad had lower odds (AOR = 0.23, 95% CI = 0.08, 0.63) of self‐reported UTI and/or BV symptoms than those who perceived water quality as good.

**Table 2:** Factors associated with any self‐reported symptoms of UTI or BV among indigenous adolescent girls.

Characteristics	Unadjusted OR (95% CI, *p* value)	Adjusted OR (95% CI, *p* value)
Age (years)		
10–15	1.00	1.00
16–19	3.26 (1.87–5.69, < 0.001)	1.85 (0.90–3.79, 0.09)
Currently studying		
Yes	1.00	1.00
No	2.13 (1.10–4.14, 0.03)	3.74 (1.23–11.39, 0.02)
Ethnicity		
Marma	1.00	1.00
Tripura	0.10 (0.05–0.21, <0.001)	0.75 (0.01–0.59, 0.01)
Tanchangya	0.15 (0.07–0.34, <0.001)	0.08 (0.03–0.27, <0.001)
Others	0.11 (0.04–0.27, <0.001)	0.07 (0.02–0.30, <0.001)
Number of family members		
<6	1.00	
≥6	0.7 (0.42–1.17, 0.17)	
Source of water for menstrual hygiene		
Underground	1.00	1.00
Surface water	1.59 (0.76–3.30, 0.22)	2.92 (1.13–7.52, 0.03)
Waterfall/spring	0.53 (0.26–1.09, 0.08)	6. 30 (1.40–28.42, 0.02)
Time required to collect water from the nearest source		
<20 min	1.00	1.00
≥20 min	0.22 (0.13–0.39, <0.001)	0.70 (0.26–1.90, 0.48)
Perceived water quality used during last menstrual period		
Good	1.00	1.00
Bad	0.18 (0.09–0.35, <0.001)	0.23 (0.08–0.63, 0.005)
Water purification to use for menstrual hygiene		
No	1.00	1.00
Yes	0.38 (0.22–0.64, <0.001)	2.22 (0.60–8.23, 0.23)
Genital wash practices		
Water and soap	1.00	1.00
Water only	2.14 (1.26–3.64, 0.005)	1.61 (0.76–3.39, 0.21)
Experienced water shortage during last menstruation		
No	1.00	
Yes	0.22 (0.13–0.38, <0.001)	0.50 (0.19–1.27, 0.14)
Frequency of genital wash per day		
≤4 times	1.00	
>4 times	1.20 (0.72–2.01, 0.69)	
Perceived difficulty score		
≤0.96 (average)	1.00	1.00
>0.96 (average)	0.25 (0.15–0.430, <0.001)	1.08 (0.41–2.88, 0.87)
Constant		0.95 (0.33–2.71, 0.92)
Pseudo *R* ^2^		0.3017

Abbreviation: OR, odds ratio.

## 4 DISCUSSION

In this study, the proportion of indigenous adolescents with any self‐reported symptoms of UTI or BV was 43.80%. The self‐reported prevalence was higher among older participants which was perhaps because they had longer environmental exposure. The odds of having any symptom was lower among those who were students than their counterparts. This is in accordance with some other studies where literate women were less prone to UTI^34^ and BV.^35^ Higher likelihood of having any symptom was associated with participants who used surface (AOR = 2.92) and spring (AOR = 6.30) water as compared with underground water users. Surface and spring water are exposed to the environment and may be contaminated by human and animal excreta and other environmental pathogens.^36^ Hence, these water sources can be reservoirs of disease‐causing agents resulting in higher likelihood to contribute to infection among users.^4^, ^5^, ^6^ For example, evidence has also shown seasonal outbreaks of waterborne diseases, such as diarrhea, in Bandarban. Low level of perceived water quality was negatively associated with any symptom of infection. The possible reason behind this association could be attributed to the fact that adolescents who perceive water quality as low may be less willing to use this water for menstruation hygiene or may treat the water before use. Although no information is available, particularly for the Chattogram Hill Tracts, purifying water through heating and filtration at household level is common in Bangladesh. For example, according to the National Hygiene Survey 2018, 14% of the households purified water before use.^37^ Likewise, lack of access to safe water and treatments required to purify water could contribute to higher perceived difficulty scores among participants. Among the participants in this study, those with higher educational attainments possessed higher perceived difficulty scores. Similar reasons could also be applied to perceived water quality. Compared with Marma, participants from Tripura, Tanchangya, Bwam, and other indigenous groups had lower odds of having any symptoms. Perhaps the sampling areas resided by Marma community had strong environmental exposure; however, existing data might not be sufficient enough to explain the other causes. Therefore, further research employing random sampling and including a big sample size is recommended.

To estimate the prevalence of UTI and BV, only self‐reported symptoms were considered. Self‐reported symptoms might result in underestimation or overestimation of the actual prevalence, however, is still an important screening tool which has been utilized by previous studies.^38^, ^39^ Furthermore, some other sexually transmitted diseases such as chlamydia, gonorrhea, trichomonas vaginalis have similar symptoms to those we considered for UTI and BV.^40^ In that case, the prevalence of the symptoms assessed may be overestimated. Before conducting the survey, however, consultations with local physicians were carried out to grasp which types of symptoms should be investigated. Indigenous populations have their own languages, and most do not have a written form. Hence, local interpreters were engaged to conduct the interviews which might also impact the data quality if interview guides were interpreted inaccurately.

## 5 STRENGTHS AND LIMITATIONS

This is the first study to observe the prevalence of self‐reported UTI and BV among indigenous adolescents during a period of water scarcity in the Chattogram Hill Tracts area of Bangladesh to our knowledge. This study presented how water scarcity, potentially due to climate change and environmental exposures, is associated with adverse adolescent menstrual health outcomes in vulnerable indigenous communities, specifically UTI and/or BV symptomatology.

In this study, convenient sampling was considered which may provide a biased estimation of self‐reported prevalence of UTI and/or BV in the population compared with that of random sampling. This sampling technique may be useful, however, when random sampling is not possible. Samples were aimed to be as representative as possible, as an attempt to ensure generalizability of this study. For example, 11 out of 29 administrative unions and 6 out of 7 administrative subdistricts were selected to conduct interviews. Regarding the predictability of the logistic regression model, the pseudo *R* ^2^ value of 0.312 indicated a moderate level of predictability.

In a multilingual and multicultural setting like Bandarban, language barriers might affect the data quality. None of the interviewers' first language was Bangla, although some of them could speak it. Some of the indigenous communities can, however, understand both Bangla and Marma languages. Translation of Bengali to other indigenous languages, such as Marma, Tripura, Tanchangya, Chak, Mro, and Bawm, might have resulted in a loss of the exact contextual meaning. The overall validity of the questionnaire was not assessed. However, items selected in the questionnaire were previously used in other settings. Although convenient sampling was employed, some of the remotest areas were not possible to cover. For example, reaching some locations such as Amiakhum and Andharmanik, requires walking through hillside streams for at least 2 days. Culturally, indigenous adolescent girls are shy and hesitate to discuss menstrual and sexual health‐related issues, therefore, this might also impact the data quality.

## 6 CONCLUSIONS

Further research is required to confirm the validity of self‐reported UTI and BV symptoms among indigenous adolescent girls in Bandarban, Bangladesh. Analytical study could be planned to observe if any episodes of UTI or BV among the study population is attributed to seasonal water scarcity in the study area.

## AUTHOR CONTRIBUTIONS

**Plabon Sarkar**: Conceptualization; data curation; investigation; methodology; project administration; resources; validation; visualization; writing—original draft; writing—review and editing. **M. A. Rifat**: Conceptualization; data curation; formal analysis; investigation; methodology; software; validation; visualization; writing—original draft; writing—review and editing. **Imdadul Haque Talukdar**: Conceptualization; data curation; investigation; methodology; validation; visualization; writing—review and editing. **Nobonita Saha**: Data curation; investigation; visualization; writing—review and editing. **Nicole S. Rodriguez Neufeld**: Visualization; writing—original draft; writing—review and editing. **Md. Ibrahim Miah**: Project administration; resources; supervision; visualization; writing—review and editing. **Sanjib Saha**: Conceptualization; supervision; visualization; writing—review and editing.

## CONFLICT OF INTEREST STATEMENT

The authors declare no conflict of interest.

## TRANSPARENCY STATEMENT

The lead author M. A. Rifat affirms that this manuscript is an honest, accurate, and transparent account of the study being reported; that no important aspects of the study have been omitted; and that any discrepancies from the study as planned (and, if relevant, registered) have been explained.

## Supporting information

Supporting information.

## Data Availability

All authors have read and approved the final version of the manuscript. The corresponding author had full access to all the data in this study and takes complete responsibility for the integrity of the data and the accuracy of the data analysis. The data are available upon reasonable request to the corresponding author.
