# Supplementary material for: Self‐reported urinary tract infection and bacterial vaginosis symptoms among indigenous adolescents during seasonal periods of water scarcity: A cross‐sectional study in Bandarban Hill District of Bangladesh
Source: Health Sci Rep. 2024 May 6;7(5):e2107. doi: 10.1002/hsr2.2107 (PMC11074625; doi:10.1002/hsr2.2107)
Supplement: Supplementary file 1 — Supporting information. [file HSR2-7-e2107-s001.pdf]

Self-reported Urinary Tract Infection and Bacterial Vaginosis Symptoms among Indigenous Adolescents during Seasonal Periods of Water Scarcity: A Cross-Sectional Study in Bandarban Hill District of Bangladesh

STROBE Statement—checklist of items that should be included in reports of observational studies

	Item No.	Recommendation	Page No.	Relevant text from manuscript
**Title and abstract**	1	(*a*) Indicate the study's design with a commonly used term in the title or the abstract	Title	See title
		(*b*) Provide in the abstract an informative and balanced summary of what was done and what was found	Abstract	See abstract
**Introduction**				
Background/rationale	2	Explain the scientific background and rationale for the investigation being reported	Paragraph 4 & 5	See paragraph 4 &5
Objectives	3	State specific objectives, including any prespecified hypotheses	Paragraph 4	See paragraph 4
**Methods**				
Study design	4	Present key elements of study design early in the paper	Section 2.2	See section 2.2
Setting	5	Describe the setting, locations, and relevant dates, including periods of recruitment, exposure, follow-up, and data collection	Section 2.1	See section 2.1
Participants	6	(*a*) *Cohort study*—Give the eligibility criteria, and the sources and methods of selection of participants. Describe methods of follow-up<br>*Case-control study*—Give the eligibility criteria, and the sources and methods of case ascertainment and control selection. Give the rationale for the choice of cases and controls<br>*Cross-sectional study*—Give the eligibility criteria, and the sources and methods of selection of participants	Section 2.2	See section 2.2
		(*b*) *Cohort study*—For matched studies, give matching criteria and number of exposed and unexposed<br>*Case-control study*—For matched studies, give matching criteria and the number of controls per case	Not applicable (N/A)	Not applicable
Variables	7	Clearly define all outcomes, exposures, predictors, potential confounders, and effect modifiers. Give diagnostic criteria, if applicable	Section 2.4	See section 2.4
Data sources/ measurement	8*	For each variable of interest, give sources of data and details of methods of assessment (measurement). Describe comparability of assessment methods if there is more than one group	*Section 2.3 & 2.4*	*See section 2.3 & 2.4*

Bias	9	Describe any efforts to address potential sources of bias	Section 2.3	See section 2.3
Study size	10	Explain how the study size was arrived at	Section 2.2	See section 2.2

Continued on next page

Quantitative variables	11	Explain how quantitative variables were handled in the analyses. If applicable, describe which groupings were chosen and why	Section 2.4	See section 2.4
Statistical methods	12	(*a*) Describe all statistical methods, including those used to control for confounding	Section 2.5	See section 2.5
		(*b*) Describe any methods used to examine subgroups and interactions	N/A	N/A
		(*c*) Explain how missing data were addressed	N/A	N/A
		(*d*) *Cohort study*—If applicable, explain how loss to follow-up was addressed<br>*Case-control study*—If applicable, explain how matching of cases and controls was addressed<br>*Cross-sectional study*—If applicable, describe analytical methods taking account of sampling strategy	Section 2.1 & 2.5	See section 2.1 & 2.5
		(*e*) Describe any sensitivity analyses	N/A	N/A
**Results**				
Participants	13*	(a) Report numbers of individuals at each stage of study—eg numbers potentially eligible, examined for eligibility, confirmed eligible, included in the study, completing follow-up, and analysed	Section 3.1	See section 3.1
		(b) Give reasons for non-participation at each stage	N/A	N/A
		(c) Consider use of a flow diagram	N/A	N/A
Descriptive data	14*	(a) Give characteristics of study participants (eg demographic, clinical, social) and information on exposures and potential confounders	Section 3.1, Table 1	See section 3.1 & Table 1
		(b) Indicate number of participants with missing data for each variable of interest	N/A	N/A
		(c) *Cohort study*—Summarise follow-up time (eg, average and total amount)	N/A	N/A
Outcome data	15*	*Cohort study*—Report numbers of outcome events or summary measures over time	N/A	N/A
		Case-control study—Report numbers in each exposure category, or summary measures of exposure	N/A	N/A
		Cross-sectional study—Report numbers of outcome events or summary measures	*Section 3.1 & 3.2*	*See section 3.1 & 3.2*
Main results	16	(*a*) Give unadjusted estimates and, if applicable, confounder-adjusted estimates and their precision (eg, 95% confidence interval). Make clear which confounders were adjusted for and why they were included	Table 2	See Table 2
		(*b*) Report category boundaries when continuous variables were categorized	N/A	N/A
		(*c*) If relevant, consider translating estimates of relative risk into absolute risk for a meaningful time period	N/A	N/A

Continued on next page

Other analyses	17	Report other analyses done—eg analyses of subgroups and interactions, and sensitivity analyses	N/A	N/A
**Discussion**				
Key results	18	Summarise key results with reference to study objectives	Paragraph 1	See paragraph 1
Limitations	19	Discuss limitations of the study, taking into account sources of potential bias or imprecision. Discuss both direction and magnitude of any potential bias	Section 5	See section 4.1
Interpretation	20	Give a cautious overall interpretation of results considering objectives, limitations, multiplicity of analyses, results from similar studies, and other relevant evidence	Paragraph 1	See paragraph 1
Generalisability	21	Discuss the generalisability (external validity) of the study results	Section 5	See paragraph 1 and 2
**Other information**				
Funding	22	Give the source of funding and the role of the funders for the present study and, if applicable, for the original study on which the present article is based	Funding	See Funding

*Give information separately for cases and controls in case-control studies and, if applicable, for exposed and unexposed groups in cohort and cross-sectional studies.
